# Role of Potassium Ions Quantum Tunneling in the Pathophysiology of Phantom Limb Pain

**DOI:** 10.3390/brainsci10040241

**Published:** 2020-04-18

**Authors:** Mustafa Alrabayah, Abdallah Barjas Qaswal, Aiman Suleiman, Lubna Khreesha

**Affiliations:** Faculty of Medicine, The University of Jordan, PO Box 13046, Amman, 11942, Jordan; m.rabayah@ju.edu.jo (M.A.); qaswalabdullah@gmail.com (A.B.Q.); lub0076@yahoo.com (L.K.)

**Keywords:** quantum neuroscience, quantum signals, pain pathophysiology, phantom limb pain, pain biophysics

## Abstract

(1) Background: multiple theories were proposed to explain the phenomenon of phantom limb pain (PLP). Nevertheless, the phenomenon is still shrouded in mystery. The aim of this study is to explore the phenomenon from a new perspective, where quantum tunneling of ions, a promising field in medical practice, might play a major role. (2) Methods: investigators designed a quantum mathematical model based on the Schrödinger equation to examine the probability of potassium ions quantum tunneling through closed membrane potassium channels to the inside of phantom axons, leading to the generation of action potential. (3) Results: the model suggests that the probability of action potential induction at a certain region of the membrane of phantom neurons, when a neuron of the stump area is stimulated over 1 mm^2^ surface area of the membrane available for tunneling is 1.04 × 10^−2^. Furthermore, upon considering two probabilities of potassium channelopathies, one that decreased the energy of the barrier by 25% and another one by 50%, the tunneling probability became 1.22 × 10^−8^ and 3.86 × 10^−4^, respectively. (4) Conclusion: quantum models of potassium ions can provide a reliable theoretical hypothesis to unveil part of the ambiguity behind PLP.

## 1. Introduction

PLP is a neuropathic pain that is felt at the site of lost body parts like limbs, eyes, breasts or visceral organs [1,2]. It is usually accompanied by other neuropathic phenomena such as stump pain and phantom sensations [3]. It is a poorly understood phenomenon [4]. Postamputation incidence ranges from 42.2–78.8% [5]. It comes in many characters, like tingling, burning, aching, needle piercing and changes in temperature sensations in a relapsing–remitting manner [6].

Quantum physics is a branch of theoretical physics. It uses mathematical models to explain natural phenomena. Quantum tunneling describes the ability of a wave particle to tunnel through a potential barrier and reach a classically forbidden area [7]. In this study, investigators designed a mathematical model to explore the quantum behavior of potassium ions in PLP. The model tests the probability of quantum tunneling of extracellular potassium ions through closed membrane potassium channels to the intracellular space of the axon, causing alterations in voltage difference, which leads to generation of an action potential. Tunneling is driven by the rise in potassium concentrations extracellularly due to adjacent neuronal hyperexcitability. 

Currently, theories of PLP target three anatomical levels: cortical level, spinal cord level and peripheral level. Many scientists believe a multilevel combination stands behind the phenomenon [4]. At the cortical level, the most cited theories are cortical remapping theory [8,9,10,11,12], neuromatrix theory [13,14,15] and proprioceptive memory theory [16,17,18]. At the spinal cord level, theories include central sensitization [19], upregulation of NMDA (N-methyl-D-aspartate) receptors in the dorsal horn of spinal cord ‘windup phenomenon’ [20] and degradation of descending inhibitory neurons from supraspinal centers [21].

Regarding the peripheral level, multiple mechanisms were proposed. After amputation, peripheral nerves are severed, causing disruption of afferent inputs to the spinal cord. This is followed by ‘deafferentation’, where the proximal portion of the injured nerve sprouts to form neuromas [22,23]. Neuromas and residual cell bodies were proposed as mechanisms for ectopic firing [24,25,26]. Upregulation of sodium channels with accompanying hyperexcitability of adjacent nerves might increase the frequency of pain attacks [27,28,29]. This is supported by a reduction in PLP bouts upon using sodium channel blockers [30]. Potassium channelopathy and downregulation were linked to multiple chronic neuropathic pain phenomena that are largely peripheral in origin [31,32,33,34]. Finally, dorsal root ganglia hypersensitivity was suggested as dorsal horn plasticity develops in response to changes in peripheral input [35,36,37,38,39,40].

Since research continues to investigate more precise mechanisms of PLP [41], our aim is to explore the phenomenon from a new perspective—quantum physics. A simple model of potassium ion quantum tunneling has been established before, and used to explain referred pain phenomenon and hyperexcitability among unmyelinated and demyelinated neurons [42], which smooths the path to apply more complex models in the explanation of PLP. Quantum tunneling is recently under heavy research due to the ability of its models to explain multiple mysterious biological phenomena [43,44] and the evolutionary medical applications that relied on it [45].

## 2. Materials and Methods

The model of quantum tunneling of potassium ions through the closed channels has been proposed [42]. This model has been used to explain referred pain [46] and understand the myelin function in limiting hyperexcitability [47]. However, in this study, the model will be used in the context and scope of the PLP phenomenon.

Potassium voltage-gated channels block the permeation of ions by forming a hydrophobic gate at the intracellular end [48]. This hydrophobic gate represents an energy barrier that prevents ions from passing through because the kinetic energy (*KE*) of potassium ions is less than the barrier energy of the hydrophobic gate (*U*). As a result, the quantum tunneling probability of potassium ions through the hydrophobic gate can be calculated.

Each type of channel has a certain value of free gating energy which represents the barrier energy (*U*) that must be overcome to open the hydrophobic gate. This barrier is illustrated as an electric field (*E*) in the space of a parallel capacitor that resists the movement of potassium ions. This electric field is calculated by the following equation [42]:

(1) $$E=\frac{U}{qL}$$

where *U* is the absolute value of gating free energy, *q* is the potassium ion charge and *L* is the length of the gate which is $4.4\times 10^{-11}$ m [42].

The tunneling probability of potassium ions through the closed gate can be calculated by the following equation [42,47,48,49]:

(2) $$P=e^{-\frac{\sqrt{8m}}{\hbar }\int_{X1}^{X2}\sqrt{qEx-KE}dx}$$

where *P* is the tunneling probability; *m* is the mass of potassium ion ($6.5\times 10^{-26}$ Kg); ℏ is the reduced Planck constant ($1.05\times 10^{-34}$ J·s); *q* is the charge of potassium ion ($1.6\times 10^{-19}$ C); *E* is the electric field that is needed to prevent the ion from passing the gate and it corresponds to the value of free gating energy, which represents the energy required to open the gate of the channel; x is the ion position through the hydrophobic gate; *KE* is the kinetic energy of potassium ion; and *X1* to *X2* is the forbidden region where the ion cannot pass through the gate.

By substituting Equation (1) in Equation (2), the following equation results:

(3) $$P=e^{-\frac{\sqrt{8m}}{\hbar }\int_{X1}^{X2}\sqrt{\frac{U}{L}x-KE}dx}$$

We then calculate the kinetic energy of extracellular potassium ions as a part of the tunneling probability equation. Extracellular potassium ions get their kinetic energy when they pass through the neuronal membrane voltage of −90 mV [50], because the hydrophobic gate is located at the intracellular end. Additionally, potassium ions get kinetic energy due to the thermal source of body temperature. Therefore, the total kinetic energy of extracellular potassium ions can be calculated by the following equation:

(4) $$KE=qV+\frac{1}{2}K_{B}T$$

where *q* is the charge of potassium ion, *V* is the neuronal membrane voltage of −90 mV, $K_{B}$ is the Boltzmann constant ($1.38\times 10^{-23}$ J/K) and T is the body temperature in Kelvin (310 K).

The forbidden region (*X1* to *X2*) is where the energy of the barrier is equal or higher than the kinetic energy of the ions as in the following equation [42]:

(5) qEx≥KE

Then, it is crucial to determine the effect of the quantum tunneling of potassium ions on the resting membrane potential. This is achieved by calculating the single channel conductance $C_{QK}$ due to the quantum tunneling [42,49,51]:

(6) $$C_{QK}=\frac{e^{2}P}{h}$$

where *e* is the electron charge, *P* is the tunneling probability and h is the Planck constant ($6.6\times 10^{-34}$ J·s).

Accordingly, the quantum membrane conductance can be calculated by the following equation

(7) $$C_{QMK}=C_{QK}\times D$$

where *D* is the channels density in the neuronal membrane and is about 5–50 channels/µm^2^ in the unmyelinated neurons [52].

## 3. Results

### 3.1. Repetitive Increases in Extracellular Potassium Concentration during Action Potential

When action potentials occur, sodium ions enter the neuron and potassium ions exit the neuron. This will increase the extracellular potassium concentration slightly, around $4.3\times 10^{-2}$ mEq/L [47]. Therefore, each time an adjacent neuron fires, there will be an increase in extracellular potassium concentration. These extracellular potassium ions associated with each action potential will tunnel through the closed potassium channels. The subsequent sections will address the calculation of tunneling probability and the quantum membrane conductance.

### 3.2. The Tunneling Probability of Potassium Ions through the Closed Gate of the Channels and Quantum Conductance

To calculate the tunneling probability of extracellular potassium ions, the energy needed to open the closed potassium channels (U) and the kinetic energy of potassium ions (KE) must be determined.

Potassium channels types Kv1.1 and Kv1.2 are located in the axons of neurons. For that reason, the calculations will be carried on Kv1.2. Kv1.2 channels need $U=5.35\times 10^{-20}$ J [47] to open their hydrophobic gate. On the other hand, by using Equation (4), the kinetic energy of extracellular potassium ions is $KE=1.65\times 10^{-20}$ J with a forbidden region from $X_{1}=1.36\times 10^{-11}$ m to $X_{2}=4.4\times 10^{-11}$ m. Consequently, by using Equation (3), the tunneling probability is $P=2.41\times 10^{-12}$.

Applying Equations (6) and (7) by substituting the potassium channels density to be 50 channels/µm^2^, $C_{QK}=9.35\times 10^{-14}$ mS and $C_{QMK}=4.68\times 10^{-4}$ mS/cm^2^.

### 3.3. The Effect of Quantum Membrane Conductance of Potassium Ions on the Resting Membrane Potential

To show the effect of the quantum membrane conductance of potassium ions on the resting membrane potential, the Goldman–Hodgkin–Katz equation is used [50]:

(8) $$V(millivolts)=-61\times \log\frac{C_{Na}{\left[Na\right]}_{i}+C_{K}{\left[K\right]}_{i}}{C_{Na}{\left[Na\right]}_{o}+C_{K}{\left[K\right]}_{o}}$$

By substituting 140 mEq/L, 4 mEq/L, 14 mEq/L, 142 mEq/L, 0.5 mS/cm^2^, and 0.005 mS/cm^2^ [42] for ${\left[K\right]}_{i}$, ${\left[K\right]}_{o}$, ${\left[Na\right]}_{i}$, ${\left[Na\right]}_{o}$, $C_{K}$ and $C_{Na}$ respectively, the resting membrane potential V=−86 mV. To determine the effect of the quantum tunneling of potassium ions associated with action potential, Equation (8) should be modified as the following:

(9) $$V(millivolts)=-61\times \log\frac{C_{Na}{\left[Na\right]}_{i}+C_{K}{\left[K\right]}_{i}}{C_{Na}{\left[Na\right]}_{o}+C_{K}{\left[K\right]}_{o}+C_{QMK}{\left[K\right]}_{AP}}$$

By substituting $C_{QMK}=4.68\times 10^{-4}$ and ${\left[K\right]}_{AP}=4.3\times 10^{-2}$ mEq/L in Equation (9), the resting membrane potential of −86 mV will not be changed because the increase in potassium concentration during action potential and the quantum conductance are both low.

### 3.4. The Threshold Value of Quantum Conductance and Quantum Tunneling to Induce Action Potential

**Figure 1:**
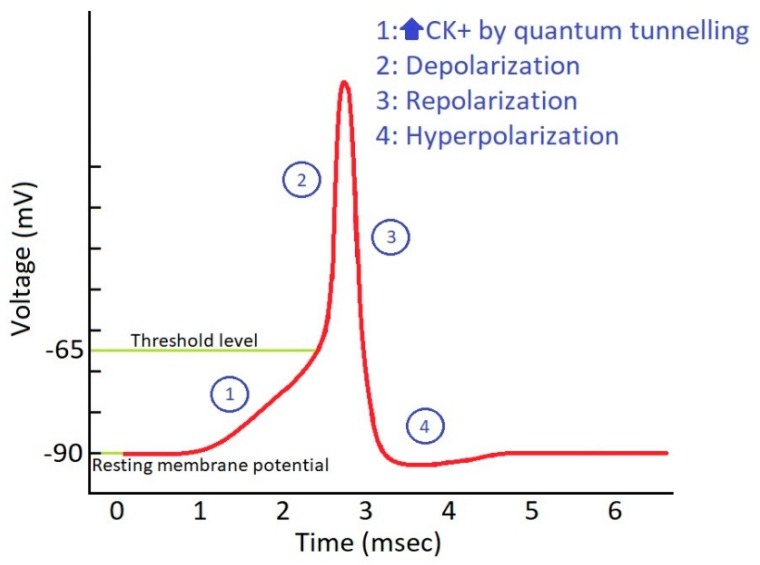
Action potential in phantom axons: role of potassium ion conductance and threshold level.

The threshold value of membrane potential that is required to induce action potential is −65 mV. By using Equation (9), the threshold value of $C_{QMK}$ is 76.92 mS/cm^2^. Assuming that this conductance is a result from one channel of the 50 channels/µm^2^, the threshold value of quantum tunneling is $1.98\times 10^{-5}$, as seen in Figure 1.

### 3.5. The Probability of Inducing an Action Potential in Phantom Limb Neurons

**Figure 2:**
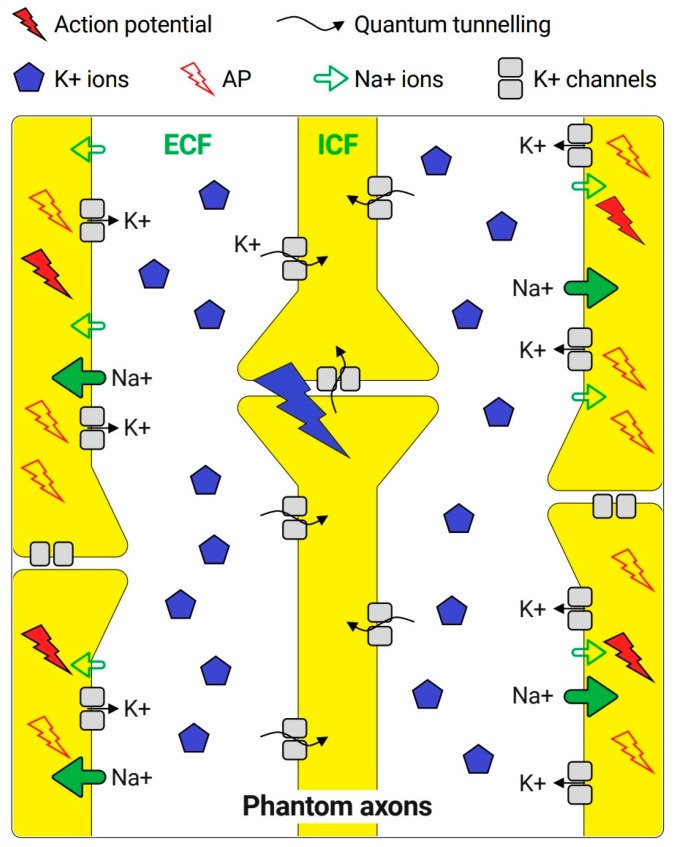
Simplifying the idea of potassium ions quantum tunneling as a consequence to hyperexcitability of adjacent nerves.

According to Section 3.3, it was found that the increase in potassium concentration during an action potential will not affect the resting membrane potential. However, when quantum tunneling occurs between neurons, there will be a probability of achieving the threshold value of quantum conductance, because there will be a sufficient number of potassium ions tunneling through the closed channels. Therefore, there will be a probability of inducing an action potential in the neurons of phantom limb by the previously hyperexcitable adjacent neurons or the neurons of stump pain via quantum tunneling, as seen in Figure 2.

This probability can be calculated by using the Bernoulli trials equation:

(10) $$P(Z)=\frac{N!P^{Z}{\left(1-P\right)}^{N-Z}}{(N-Z)!Z!}$$

where N is the total number of trials, Z is the number of successful trials, *P* is the probability of success, and *P*(*Z*) is the probability of achieving a certain number of successful trials. For example, if *Z* = 0, then the equation will be:

$$P(0)={\left(1-P\right)}^{N}$$

The following steps must be considered to calculate the probability of inducing action potential in phantom limb neurons:

1. When action potential happens, there will be $4.36\times 10^{3}$ ions/µm^2^ [48] and there are 50 channels/µm^2^. Therefore, on average, there will be N=87 potassium ions trying to tunnel each channel [47]. Additionally, the tunneling probability, which represents the probability of success in passing the closed channel (P), is $2.41\times 10^{-12}$. However, in section D, the threshold tunneling probability to induce an action potential is $1.98\times 10^{-5}$ which also represents the sufficient fraction of potassium ions that must tunnel to induce action potential. Therefore, if one of the 87 potassium ions tunneled, there will be a fraction of 1/87 = $1.15\times 10^{-2}$, which is higher than threshold, and will be enough to induce an action potential. Therefore, the probability that at least one of the 87 potassium ions will tunnel through the closed channel is:
  $$\begin{array}{l} P_{a}=1-P(0)\\ P_{a}=1-{\left(1-2.41\times 10^{-12}\right)}^{87}\\ P_{a}=2.1\times 10^{-10} \end{array}$$
2. Furthermore, for the sake of simplicity, it is assumed that one channel is enough to induce the action potential. Therefore, the probability that at least one channel from the 50 channels/µm^2^ in the membrane of unmyelinated neurons will be tunneled by a sufficient fraction of potassium ions is calculated as the following:
  $$\begin{array}{l} P_{b}=1-P_{a}(0)\\ P_{b}=1-{\left(1-2.1\times 10^{-10}\right)}^{50}\\ P_{b}=1.05\times 10^{-8} \end{array}$$
3. Finally, the probability will depend on the surface area available for quantum tunneling; the larger the surface area, the higher the probability of inducing an action potential. For a surface area of 1 mm^2^, there will be 10^6^ times of (1) µm^2^. If one of these areas was tunneled by a sufficient fraction of potassium ions, action potential will be induced and propagated along the axon to be transmitted to the brain and perceived as PLP. Therefore, stimulation of at least one area will be enough to transmit the action potential to the brain. Thus, the probability that at least one area is stimulated by quantum tunneling is:
  $$\begin{array}{l} P_{c}=1-P_{b}(0)\\ P_{c}=1-{\left(1-1.05\times 10^{-8}\right)}^{10^{6}}\\ P_{c}=1.04\times 10^{-2} \end{array}$$

This final value represents the probability of action potential induction at a certain region of the membrane of phantom limb neurons when a neuron of the stump area is stimulated with 1 mm^2^ surface area of the neuronal membrane available for tunneling.

Adding to this, important considerations should be made. Quantum tunneling between unmyelinated neurons is more obvious and evident unlike myelinated neurons where potassium channels are covered by a thick layer of myelin eliminating the quantum tunneling of potassium ions through the closed channels [47]. Therefore, demyelinated neurons at the site of injury will get the opportunity of potassium tunneling and action potential induction because in this case demyelination exposes closed channels to potassium ions. As a result, the demyelinated part will carry the action potential signals to the myelinated part of the same neurons, generating a state of hyperexcitability among myelinated neurons such as the neurons of fast pain (Aδ fibers).

**Figure 3:**
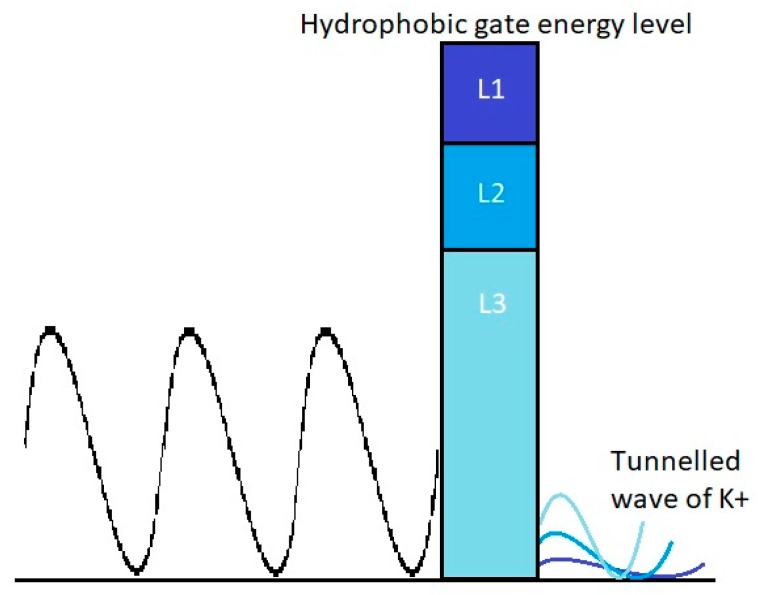
Effect of channelopathy on free gating energy and potassium ion quantum tunneling.

Another important factor that plays a major role in predisposition to phantom pain is channelopathy. When channelopathy occurs, it might decrease the energy barrier of the closed channels (U) and this will increase the tunneling probability and consequently the probability of inducing action potential in the neurons of phantom limb, as seen in Figure 3. The previous calculations were based on that of the energy barrier $U_{1}=5.35\times 10^{-20}$. However, if two potassium channelopathies occurred, one decreased the energy barrier by 25% so that it became $U_{2}=4.01\times 10^{-20}$ J, and the other decreased the barrier by 50% so that it became $U_{3}=2.68\times 10^{-20}$ J, the tunneling probability will become $1.22\times 10^{-8}$ and $3.86\times 10^{-4}$, respectively. Additionally, by following the previous 3 steps, the probability of action potential induction for both cases of channelopathies is almost 100%. See Figure 3.

## 4. Discussion

### 4.1. Scope and Rationale

The role of the somatosensory cortex in causing phantom pain is greatly debated. Although studies pointed to the potential role of cortical remapping [53,54], recent series of experiments showed no significant relationship between cortical reorganization and PLP, arguing that multiple different factors may play a role in maintaining the functional abilities of the brain to control phantom pains [55]. No neuropathic pain was produced following extensive electrical stimulation of the remapped brain areas in the somatosensory cortex [56]. Going further, a recent study found that increasing the phantom representation cortically was associated with increased pain, opposing the assumptions of cortical remapping theory [57].

We relay our focus on peripheral mechanisms as they come with more supporting evidence [4]. Neuraxial anesthesia and peripheral nerve blocks were proven to decrease the severity and the frequency of PLP postoperatively [58]. Anesthetizing the residual limb by local anesthetic injections is reported to attenuate PLP for weeks [59,60,61]. Nevertheless, not all cases reported abolishment of pain, emphasizing the multilevel approach to the phenomenon [59]. More recent studies on the peripheral causes of PLP focused on the inability of severed nerves to repair previous connections and the role of preamputation pain and stump pain [62].

Phantom axon or phantom neuron represents the remaining proximal part of the neuron that was supplying the limb before amputation. In our model, we were concerned about the source of the triggered action potentials in phantom axons, as receptive fields were lost during amputation. Neuromas were suggested before, but have been debated by many experimental studies that showed disappointing results upon neuromas ablation [63,64,65,66]. The question this study aims to answer is: how can hyperexcitability of nerves at the stump region (due to pain preoperatively or stump pain postoperatively), along with neuronal degeneration and potassium channelopathy, be collected in a picture that explains PLP?

Based on collective understanding of the literature, the ability to prove that the quantum tunneling of potassium ions may play a significant role in PLP depends on our ability to construct four pillars: (1) nerves at the stump region are at a hyperexcitable state, where pain preoperatively triggers hyperexcitability and stump pain postoperatively maintains it; (2) quantum models suggest probabilities, hence, pain should decay overtime postoperatively as hyperexcitability of adjacent neurons decay, have a relapsing-remitting manner that depends on probability succession and come in multiple characteristics that could be carried by unmyelinated or demyelinated axons; (3) nerves at the stump region should exhibit upregulation of sodium channels with higher spontaneous discharges, which will cause intermittent rises in extracellular potassium concentrations due to higher rates of depolarizations; (4) nerves that were severed during amputation will exhibit membrane channelopathies due to aberrant expression of channel proteins, which will alter the hydrophobicity of the channel gate, thus decreasing the energy of the barrier [67].

Pain in the perioperative period is thought to be the most important cause of oversensitivity and hyperexcitability of the amputated limb nerves [68]. Studies showed that people who had preamputation pain suffered higher rates of PLP [69,70,71]. Stump pain, which is pain at the residual part of the amputated limb, has a significant positive correlation with PLP [72,73]. PLP occurs more frequently if stump pain stays for longer periods post-operatively, and may subside when stump pain subsides [74].

PLP starts a few days postoperatively [70], which stands against the role of neuromas and emphasizes the role of hyperexcitability. It also decays over time postoperatively, which matches the previous assumption, as hyperexcitability of adjacent nerves decays overtime [75,76]. The model supports the intermittency of pain, as pain depends on succession of the tunneling probability of potassium ions, hence, PLP must occur in a relapsing–remitting manner [70]. Succession of probability along unmyelinated and demyelinated fibers explains the multiple characters of PLP like burning, stabbing, itching and heaviness [62,77]. In unmyelinated C fibers, potassium channels are already exposed, while in myelinated Aδ fibers, nerves undergo Wallerian demyelination following injury, which is a process that exposes potassium channels on the membrane, leading to varieties of painful sensations [78]. In addition, pain is mostly localized to the distal portion of the phantom limb (fingers in upper limbs, toes in lower limbs) [62], which further emphasizes that the action potentials conveying pain are generated in phantom axons.

In regard to ion channels, there are many adaptive processes that accompany nerve injury, inflammation or degeneration, all of which might contribute to major phenotypic changes, which can boost the excitability of peripheral nociceptors. Medium to long-term changes can be attributed to transcriptional and epigenetic regulation of ion channel gene expression, changes in trafficking, and post-translational modifications [33]. Minutes following amputation, central sensitization takes place [19]. This is followed by major alterations in channels, including upregulation of voltage-gated sodium channels [79,80,81,82], which sensitizes the receptive fields [79]. Upregulation of sodium channels plays a major role in neuropathic pain and is strongly suggested as the mechanism behind neuronal hyperexcitability [27,28,29,30]. Nevertheless, an undoubtful causality between sodium channels upregulation and hyperexcitability has never been established. A study held on mice neuromas showed little evidence of association between altered levels of sodium channel α-subunit protein and nerve hyperexcitability [83]. Another study proved the ability to develop normal levels of neuropathic pain in mice that lack Nav1.3 channels [84]. These studies strongly suggest that the mechanism is beyond the scope of channel individuality, and hyperexcitability should trigger a series of following events that need to be discussed.

Potassium channels might play a major role in evoked and spontaneous hyperexcitability of the severed nerves [33]. Theoretically, it seems that inhibition of potassium channels will prolong depolarization phase and thus decrease the action potential frequency, but in the majority of cases, potassium channels downregulation increased the frequency of action potentials [85]. This is supported by multiple studies showing that the inhibition of potassium channels with broad spectrum potassium channel blockers induced spontaneous activity in the nerve fibers [31,32]. Recently, a study conducted in the Mayo Clinic on patients with known autoantibodies against voltage-gated potassium channels found that pain was reported in 50% of cases, which is five times more frequent than pain incidence in patients with other types of neurological autoantibodies [34]. The only neuropathology found in these patients was abnormal cutaneous nociceptive fibers [34]. This supports the idea that pain related to potassium channelopathy is largely peripheral in origin, and that downregulation of potassium channels can be a contributing factor in pain syndromes [33]. Multiple experimental studies found that genetic mutations of genes coding for potassium channel subunits have significant association with neuronal overexcitability in neuropathic pain [33]. Research on animals supports the idea that aberrant expression and downregulation of potassium channel subunits are characteristic features of nerves that convey neuropathic pain [86,87]. The majority of current research focuses on depolarizing ion channels like sodium and calcium channels, while research on potassium channels is less abundant [33]. Channelopathy that accompanies downregulation decreases trafficking across channels and renders them dormant, but measuring the exact figures of kinetics around potassium channels is difficult due to the large heterogenicity of the channels and large number of channels that might be involved [33].

### 4.2. Limitations

We realize our model is theoretical, as it is based on theoretical physics. In a sense, it is currently impossible to test quantum tunneling directly, as placing any kind of detector inside a barrier would effectively change the form of the potential [88]. Accurate measurement of kinetics surrounding the potassium channels is labelled difficult as there are more than fifteen types of potassium channels involved in pain perception, in addition to the intermittency of PLP [33]. Literature lacks many figures that are important to apply more complex quantum models on PLP. We realize that experimental evidence with accurate figures regarding potassium kinetics is needed to link our hypothesis to PLP with high confidence. The model can add significantly to the knowledge of PLP, but to a lesser extent to other phantom sensations that rely entirely on myelinated neurons.

Other challenges include the presence of PLP in people with congenital absence of limbs [80], and pain that might disappear or change in severity and frequency overtime when using prosthesis [76]. The incidence of PLP in the congenital group is 3.7%, compared to 48.5% in the surgical group [89]. In addition, there are reported cases of failure of local anesthetic injection to remit phantom pain, which adds to the challenges facing theories that rely entirely on the peripheral level [8]. A balanced view of phantom limb sensations is needed to explore the phenomenon from all aspects and build a harmony between theories [90]. Finally, quantum models of potassium ions can be an area of research in any neuropathic pain, but the specific surrounding conditions made the probability extremely significant in PLP.

## 5. Conclusions

The probability of action potential induction at a certain region of the membrane of phantom limb neurons, when a neuron of the stump area is stimulated within 1 mm^2^ surface area of the neuronal membrane available for tunneling, is 1.04 × 10^−2^. This probability can reach up to 100% when applying the suggestion of channelopathy. The characteristics and specifics of PLP can be explained in the scope of potassium ion quantum tunneling. We recommend further investigations and clinical research targeting the hyperexcitability of adjacent nerves and the fidelity of potassium channels at the severed regions to optimize the approach to PLP.
